# Robotic-assisted aberrant thoracic duct ligation for persistent chyle-leak post cervical rib resection

**DOI:** 10.1093/jscr/rjad005

**Published:** 2023-01-19

**Authors:** Amalan Thuraisingam

**Affiliations:** Department of Cardiothoracic Surgery, Monash Medical Centre, Monash Health, Clayton 3168, Victoria, Australia

## Abstract

Chyle leak following cervical rib resection is a serious uncommon complication. Aberrant anatomy can further confound treatment and require the involvement of multiple specialties to delineate the ducts course and allow successful management.

## INTRODUCTION

Neurogenic thoracic outlet syndrome (nTOS) presents both a diagnostic and management challenge for surgeons. Open surgical thoracic outlet decompression can be highly effective for the treatment of nTOS, however it does carry significant risks. Chyle leak is a particularly feared complication of thoracic outlet surgery, with a reported rate of 1–1.5% [1]. Aberrancy of the thoracic duct anatomy does exist and our case report will highlight the importance of recognizing these variations and management options in the event of persistent chyle leak.

## CASE REPORT

A healthy 25-years-old female was referred to our vascular team with a history and clinical examination suggestive of left-sided nTOS. The patient had been experiencing a 10-month history of significant left-sided shoulder and neck pain that had a radicular component to medial upper arm and forearm. She had bilateral cervical ribs and severe exacerbation of her symptoms when the arms were placed above her head.

A magnetic resonance imaging scan did not reveal any significant cervical canal stenosis nor foraminal stenosis. Her nerve conduction study indicated reduced amplitude in the left medial antebrachial and ulnar sensory responses. After failed conservative measures, a decision was made to proceed Surgical Thoracic outlet decompression.

A left-sided transverse supra-clavicular approach was made. The cervical rib and a tight broad musculo-fascial band that extended from it to scalene tubercle of the first rib were identified. Division/ resection of this fascial band was performed, and the cervical rib was resected using a bone nibbler. Anterior scalenectomy, middle scalenotomy and plexus neurolysis were also performed. After these manoeuvres, the plexus was noted to be decompressed so the first rib was not removed. Co-seal was applied to the lymphatic bed, and a Blake’s drain was inserted.

On Day 1 postoperatively, 850 ml of obvious chylorrhea was observed in the drain. The patient commenced on the low-fat diet and octreotide 100 μg tds. On Day 3, the neck wound was re-explored via the original incision site due to an ongoing drainage. An active leak was identified and ligation of communicating channels was performed. The patient was continued on low-fat diet. Drainage declined to 250 ml per day but then rapidly increased.

The cardiothoracic team was consulted on Day 5 post-re-exploration. Despite being kept nil by mouth and receiving total parenteral nutrition, the patient continued to have a significant chyle leak—>1000 ml per day. She was taken to theatre on Day 13 for a right-sided robotic-assisted thoracoscopy thoracic duct ligation. The duct could not be found despite a diligent and prolonged exploration, high volume drainage continued.

**Figure 1:**
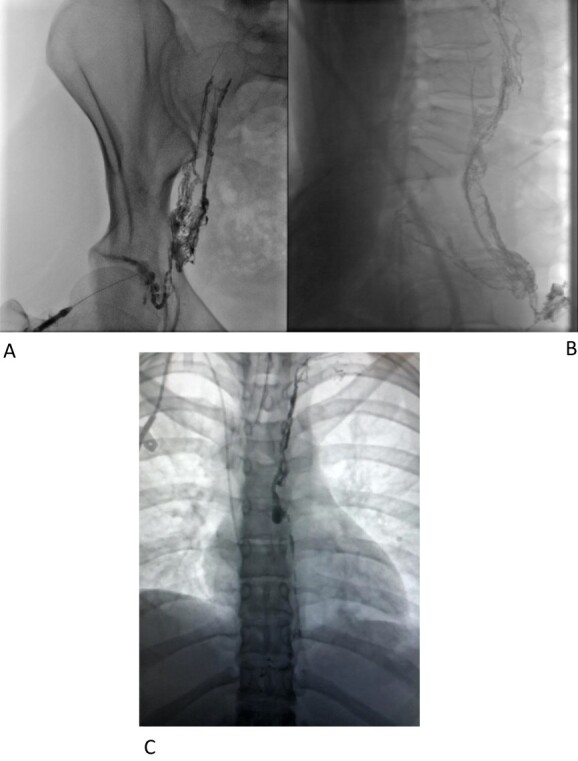
(**A**–**C**) Intranodal lymphangiogram.

Interventional radiology was consulted for possible endovascular embolization of the thoracic duct. An intranodal lymphangiogram was performed by insertion of a needle into a right inguinal lymph node and a slow injection of Lipiodol (Fig. 1a and b). The contrast passed through the pelvis and right side of the retroperitoneum to the level of L2–L3. At this level, the contrast passed to the left side via multiple tiny collaterals. No cisterna chyli was identified, preventing this avenue to cannulation/embolization. A small calibre thoracic lymphatic duct opacified in the left paravertebral region that communicated with a focal dilatation at the level of T7. Superior to this, the lymphatics were duplicated for a short segment before reuniting (Fig. 1c) then expelling Lipiodol into the drain tube. An attempt was made to cannulate the cervical thoracic duct percutaneously under ultrasound and fluoroscopic guidance, but this could not be achieved.

Immediate supplementary CT was performed whilst the Lipiodol was in situ to gather as much three-dimensional information as possible. Worryingly the patient developed positive blood cultures for candida glabrata and commenced on Anidulafungin.

**Figure 2:**
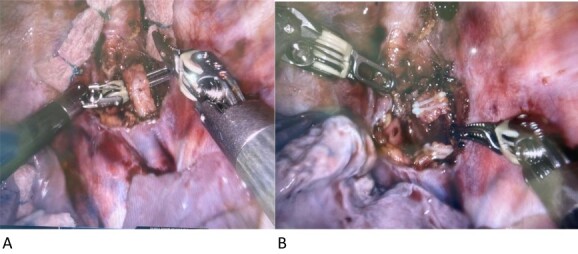
(**A** and **B**) Intraoperative left-sided thoracic duct.

Three days post lymphangiogram, the patient was returned to theatre for ligation via the left side of the chest. A high-fat meal was given to the patient 6 h pre op to facilitate visual identification of chyle containing structures. As per the CT scan, the thoracic duct was identified postero-lateral to the left subclavian artery. A short segment was excised and confirmed on frozen section (Fig. 2a and b). Rapid resolution of the lymph leak occurred within 48 h. The patient commenced on a full ward diet on Day 30 from her initial surgery and discharged home on Day 48 after fungaemia cleared.

### Follow-up

On follow-up 7 weeks post surgery, the patient had no lymphatic discharge, no lymphocele or chylothorax. The pain in her arm and forearm had resolved; however, she continued to experience pain in her trapezius.

### Comment

Chyle leak occurs in thoracic outlet decompression in the supraclavicular approach due to damage to the thoracic duct or its tributaries. The duct commences at the upper end of the cisterna chyli in the abdomen. It enters the thorax through the diaphragmatic aortic opening and usually passes upwards to the aorta’s right and comes to lie against the right side of the oesophagus. As it inclines up to the left, alongside the aorta, it passes behind the oesophagus to reach its left side at the superior mediastinum. The thoracic duct finally arches forwards across the left pleura’s dome to enter the point of confluence of the left internal jugular and subclavian veins [1, 2]. Reported anatomical variations occur in about 35% of cases [3]. In our patient, there was no cisterna chyli identified within the abdomen and the duct was not on the right side. In retrospect, if we have confirmed the presence of aberrant anatomy, then the initial thoracic duct ligation would have begun on the left side not the right.

Conservative management may settle the chyle leak in about 50% of cases. Chyle leak can be broadly categorized as low or high output with 500 ml/day being the cut off [4]. A surgical option should be considered when there is a significant chyle leak [1, 5].

Inability to locate the thoracic duct can lead to a delay in definitive treatment, poor wound healing, malnutrition and immunosuppression. An initial attempt was made via the right side of the chest based on the assumption of normal anatomy. Multiple previous reports have successfully approached the duct via a right-sided thoracoscopic approach at the diaphragmatic hiatus between the oesophagus and the azygous vein [4, 5].

After this approach failed, clear delineation of the duct course was sought via lymphangiography. A left-sided approach was made, and the thoracic duct was successfully ligated using robotic-assisted thoracoscopy.

## CONCLUSION

Although a rare complication of thoracic outlet surgery, chyle leaks are associated with high levels of morbidity and potentially mortality. Lymphangiography in the event of the development of chyle leaks facilitates recognition of thoracic duct aberrancy and subsequently guides prompt definitive treatment.
